# Hypoxia and Inflammation as a Consequence of *β*-Fibril Accumulation: A Perspective View for New Potential Therapeutic Targets

**DOI:** 10.1155/2019/7935310

**Published:** 2019-06-26

**Authors:** Matteo A. Russo, Carlo Tomino, Enza Vernucci, Federica Limana, Luigi Sansone, Andrea Frustaci, Marco Tafani

**Affiliations:** ^1^MEBIC Consortium, San Raffaele Rome Open University, Via val Cannuta 247, 00166 Rome, Italy; ^2^Department of Cellular and Molecular Pathology, IRCCS San Raffaele, Via val Cannuta 247, 00166 Rome, Italy; ^3^IRCCS San Raffaele, Scientific Direction, Via Val Cannuta 247, 00166 Rome, Italy; ^4^Department of Cardiovascular, Nephrologic, Anesthesiologic and Geriatric Sciences, Sapienza University of Rome, Viale Regina Elena 324, 00161 Rome, Italy; ^5^Laboratory of Cellular and Molecular Pathology, IRCCS San Raffaele Pisana, San Raffaele Open University, Via Val Cannuta 247, 00166 Rome, Italy; ^6^Laboratory of Cellular and Molecular Cardiology, IRCCS “L. Spallanzani”, Via Portuense 292, 00149 Rome, Italy; ^7^Department of Experimental Medicine, Sapienza University of Rome, Viale Regina Elena 324, 00161 Rome, Italy

## Abstract

Amyloidoses are heterogeneous diseases that result from the deposition of toxic insoluble *β*-sheet fibrillar protein aggregates in different tissues. The cascade of molecular events leading to amyloidoses and to the related clinical manifestations is not completely understood. Nevertheless, it is known that tissue damage associated to this disease involves alteration of tissue architecture, interaction with cell surface receptors, inflammation elicited by the amyloid protein deposition, oxidative stress, and apoptosis. However, another important aspect to consider is that systemic protein massive deposition not only subverts tissue architecture but also determines a progressive cellular hypertrophy and dilation of the extracellular space enlarging the volume of the organ. Such an alteration increases the distance between cells and vessels with a drop in pO_2_ that, in turn, causes both necrotic cell death and activation of the hypoxia transcription factor HIF-1*α*. Herewith, we propose the hypothesis that both cell death and hypoxia represent two important events for the pathogenesis of damage and progression of amyloidoses. In fact, molecules released by necrotic cells activate inflammatory cells from one side while binding to HIF-1*α*-dependent membrane receptors expressed on hypoxic parenchymal cells on the other side. This latter event generates a signaling cascade triggering NF*κ*B activation and chronic inflammation. Finally, we also suggest that this scenario, once proved and detailed, might suggest important targets for new therapeutic interventions.

## 1. Introduction

Amyloidoses are a group of heterogeneous diseases presenting many common molecular, cellular, and clinical features strictly associated to a shared pathogenetic mechanism of the tissue/organ damage [1].

Although the available clinical descriptions and studies of different amyloidoses are numerous and several studies have detailed the molecular characteristics of the proteins and their aggregates in the disease, the precise molecular pathogenetic mechanism that leads to the tissue damage is still incomplete and debated [2].

Recently, a number of authors have suggested an important role for inflammation, both as a trigger of amyloidoses and as a consequence of the *β*-fibril formation and accumulation [3, 4]. In addition, inflammation has been identified as an independent negative prognostic factor for clinical progression and severity [5–7]. However, very few studies have considered and explored the hypothesis that hypoxia, due to *β*-fibril accumulation, might represent an important pathogenetic factor by favoring the inflammatory-reparative response (IRR) and the consequent cellular damage [3, 4, 8].

The main focus of this short perspective review is to highlight the early and substantial role of hypoxia and hypoxia-triggered inflammation in producing tissue damage observed in the progressive advanced amyloidoses.

## 2. Molecular Features of Amyloidoses

**Figure 1:**
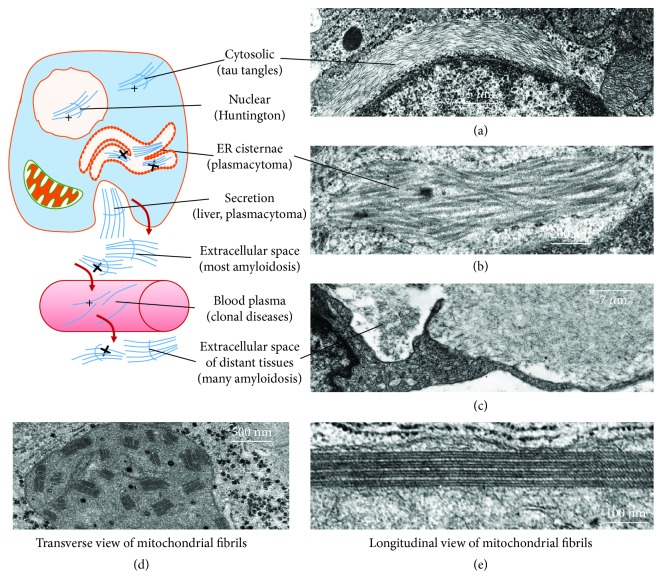
Production and localization of *β*-fibrils. The drawing shows the intracellular and the extracellular localization of *β*-fibrils of amyloidosic substance as observed at the electron microscope in different *β*-fibrilloses. Transmission electron micrographs of *β*-fibril localization in the cytosol (tau tangles of Alzheimer's disease) (a), in the cisternae of the endoplasmic reticulum (light chain polymers in a transformed B-lymphocyte/plasma cell) (b), and in the extracellular space (c) in close contact with a macrophage in the process of phagocytizing the fibrillar amyloid substance. Occasionally, *β*-fibrils have been observed in the mitochondrial matrix, being frequently organized as paracrystals (d, e). The nature of fibrillar protein is usually unknown. Mitochondria bearing fibril accumulation usually increase their volume, suggesting that accumulated misfolded fibrillar protein can be either imported from the cytosol or endogenously synthesized by mitochondrial machinery.

A constant feature of amyloidoses is the progressive accumulation of *β*-fibrils in the intra- and extracellular space of involved tissues and organs as reported in Figure 1.

In particular, *β*-fibrils can also accumulate in the blood plasma. We have termed such an accumulation as “clonal diseases.” In fact, “clonal diseases” are pathologic human conditions characterized by proliferation of a single cell lineage, mostly referred to disorders of the immunohematologic diseases, such as plasmocytoma/multiple myeloma [9–11]. In this case, patients typically present with bone marrow infiltration of *clonal* plasma cells and *monoclonal protein* in the serum and/or urine. Occasionally, when polymers are more than one, “clonal peaks” may be more than one. Clinical pathologists refer to clonal diseases (maybe inexactly) when abnormal “clonal” peaks are evidenced in serum protein electrophoretic trace, for instance, when abnormal production of a single protein occurs (liver, kidney, producing adenomas, etc.). In the case of *β*-fibrilloses, the “abnormal clonal peak” may be evidenced in the serum and/or urine not only in the case of plasma cell disorders but also in other conditions in which abnormal production of a single protein can give rise to *β*-fibrils [11].

Nevertheless, *β*-fibrils can also accumulate intracellularly in various cell compartments (Figure 1) [1].

*β*-Fibrils are polymers of *β*-sheet-rich proteins, which in normal conditions and conformation accomplish specific cell functions. Upon mutations or abnormal posttranslational modifications, they undergo misfolding, polymerization, loss of function, and possibly, acquisition of toxicity [12].

Initial protofibrils are small polymers of antiparallel *β*-sheet-rich misfolded proteins. Larger polymers are linear, rigid, and nonbranching, with an 8-12 nm diameter, easily detectable under a transmission electron microscope (TEM) (Figure 1) [1, 2, 12]. *β*-Fibrils exhibit a green birefringence at polarized light under an optical microscope after red Congo staining and more importantly a strong resistance to proteases that normally provide to their turnover in order to avoid accumulation in cells and tissues [2].

*β*-Fibrils are heterogeneous in their origin, localization, and composition, depending on the involved proteins (Table 1).

**Figure 2:**
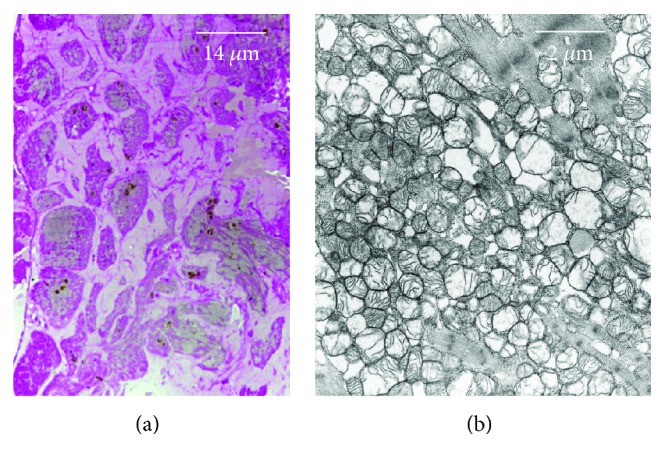
Aspects of cardiac amyloidosis with accumulation of mutated transthyretin amyloid (a). The extracellular space (ES) is constantly enlarged increasing the distance between myocardiocytes and vessels (not visible). (b) At TEM, myocardiocytes display mitochondria with various degree of swelling and alteration of cristae; both are early cell reactions to ATP depletion and hypoxia. Sarcomere ultrastructure is still intact.

**Figure 3:**
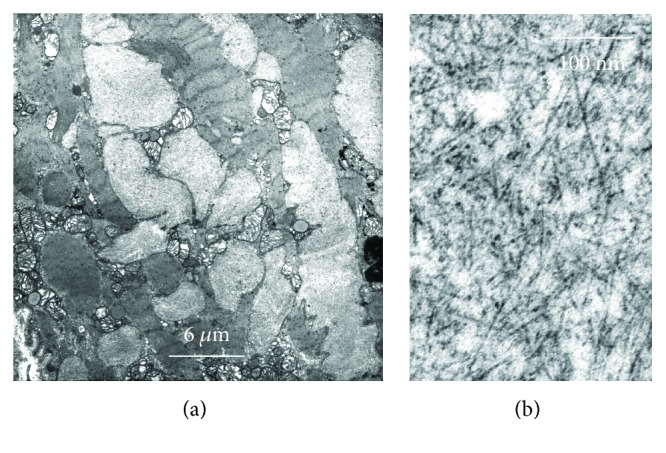
Aspects of myocardial atrial amyloidosis with intracellular accumulation of *β*-fibrils constituted of polymerized ANP (atrial natriuretic factor) peptides. (a) An advanced disorganization of the cytoplasm is evident due to the displacement of the various cytoplasmic components. (b) High-magnification detail of 10 nm *β*-fibril aggregate.

Fibrillar aggregates can be found in different subcellular compartments (Figure 1) or in extracellular space (Figure 2), where they can be easily detected because of their birefringence. Intracellular *β*-fibrils are also detectable by TEM as *cytosolic* bundles or aggregates in Alzheimer's disease (such as tau protein tangles) (Figure 1(a)), Parkinson's disease frontotemporal dementia, and dementia with Lewy bodies (*α*-synuclein and tau protein) and as *nuclear* aggregates, in Huntington's disease and other polyglutamine expansion diseases [1, 13]. Occasionally, *β*-fibrils can be observed in other subcellular compartments such as mitochondria, autophagosomes, and *cisternae of the endoplasmic reticulum* (Figure 1(b)) [14, 15]. The localization in the cisternae of the endoplasmic reticulum is more frequent in systemic amyloidosis where misfolded and polymerized proteins accumulate in producing cell, such as B lymphocyte/plasma cell (antibody light chain), pancreatic islet *β*-cell (insulin), and atrial myocardiocyte (ANF or atrial natriuretic factor) (Figure 3) [16].

Interestingly, sometimes, secretion of *β*-fibrils can be observed at the secretory pole of the cell producing the involved protein. Intracellular *β*-fibrils are able to activate various pathways of oxidative metabolism with production of ROS which contribute substantially to the cell damage.

The misfolded proteins, once accumulated outside the cell, can become *resident* in the extracellular space (Figure 1(c)), probably fixed by some interaction with common domains of various components of the extracellular matrix. In particular, this interaction may involve glycosaminoglycan- (GAG-) rich components, such as fibrillar (collagens, elastin, laminins, and fibronectins) and nonfibrillar glycoproteins (proteoglycans, hyaluronans) [17]. In alternative, misfolded proteins or their polymers can reach the *circulation* by entering the vessel lumen. This latter situation has two consequences: (a) the protein can be evidenced as clonal peak in serum protein electrophoretic trace and (b) it can infiltrate and deposit in the extracellular space in distant organs and tissues.

Interestingly, in the first case, proteins and their polymers can be appropriately measured for diagnosis and disease monitoring [18]. Moreover, in systemic amyloidoses, 15% of amyloidosic substance is constituted by SAP or *serum amyloid protein*, a member of the major acute-phase protein family, i.e., pentraxins, which strongly interacts with *β*-fibrils, probably through chemical patterns usually recognized by pentraxins. This interaction may protect *β*-aggregates from proteolysis on one side and stimulate their phagocytosis by macrophages on the other side (Figure 1) [19, 20].

## 3. Clinical Features of Amyloidoses

Amyloidosis can be *familial* or *acquired*. In the first case, numerous mutations of the involved proteins have been described, strictly influencing the misfolding, the instability, and the propensity to form *β*-fibrils [2]. *Acquired* amyloidosis is generally secondary to conditions presenting abnormal/prolonged production and secondary misfolding of involved protein. Typical examples are represented by the excessive production of Ig light chain (lambda or kappa) by a transformed clone of lymphocyte/plasma cell and by the abnormal production of acute-phase proteins such as Serum amyloid A (SAA) or transthyretin in chronic inflammatory diseases and ageing [1, 2, 21].

*β*-Fibrillosis can be *localized* or *systemic*. *Localized or organ-limited amyloidoses* accumulate, both intracellularly and in the extracellular space, polymers of specific proteins such as *β*-amyloid (neurons in Alzheimer's disease), atrial natriuretic factor (in atrial myocardiocytes), procalcitonin (transformed medullary thyroid cells), and amylin (in pancreatic islet cells). *Systemic* amyloidoses are characterized by the deposition of culprit proteins such as immunoglobulin light chains (intact or fragments) and many acute-phase proteins (SAA, transthyretin, fibrinogen *α*-chain, *β*_2_-macroglobulin, lysozyme, and gelsolin) [1, 21]. Upon entering the vessels, culprit proteins and their polymers may be evidenced in the electrophoretic diagram of blood plasma as a narrow high peak or by different bands when polymers of different MW are present. After leaving the blood, *β*-fibrils accumulate in the extracellular space of distant organs (liver, kidney, myocardium, lungs, brain, etc.), such as primary systemic amyloidosis (Ig light chains monomers and polymers or macroglobulins) and senile systemic amyloidosis (nonmutated transthyretin monomers), triggering the variable clinical features, typical of systemic amyloidoses [22].

*β*-Fibrilloses are *progressive* diseases in relation to the rate of accumulation of *β*-aggregates and to the severity of specific organ dysfunctions, as it occurs in Alzheimer's or Parkinson's disease, in myocardial amyloidosis by transthyretin and in kidney amyloidosis in the course of primary systemic amyloidosis [13, 23–25].

*“False hypertrophy”* of the involved organ/tissue, as evaluated by imaging, may be the first clinical sign for a diagnosis of amyloidosis [23]. *β*-Fibrils accumulate in the parenchyma of large organs, including the liver, kidneys, lungs, myocardium, skeletal muscle, intestine, and brain, progressively subverting their architecture and function [26, 27]. Typically, tissues show enlarged extracellular spaces, occupied by the amyloid substance (apparently amorphous and similar to the starch) (Figure 2(a)), which, as said, under optical microscope (OM) exhibits a green birefringence at polarized light after red Congo staining while under TEM appears composed of *β*-fibrils, 8-12 nm in diameter (Figure 3(b)) (Tables 1 and 2).

### 3.1. Hypoxic Microenvironment Generation

In tissues accumulating *β*-aggregates, the distance between vessels and parenchymal cells progressively increases above 200 *μ*m representing the physical limit for an efficient gas diffusion and exchange (oxygen from blood and carbon dioxide from cells) [3]. As a consequence, in this area, a hypoxic environment is generated, leading to HIF-1*α* activation that is accompanied by NF*κ*B activation. Importantly, both HIF-1*α* and NF*κ*B trigger and amplify the inflammatory-reparative response and cellular damage [28].

### 3.2. Consequences of HIF-1*α*/NF*κ*B Axis Activation

#### 3.2.1. Hypoxic Cell Damage and Cell Adaptation to Hypoxia

*Acute* hypoxia causes cell death. O_2_ shortage causes inhibition of ATP production, rapid fall of the energy charge, and loss of ionic gradients with the alteration of cytosolic calcium homeostasis [29]. The increase of cytosolic Ca^++^ concentration above 10^−6^ M dramatically activates the peroxidative metabolism, an irreversible contraction and degradation of the cytoskeleton and the activation of Ca^++^-dependent cytosolic proteases (calpains) and DNAases, leading to an irreversible and rapid cell degradation [30]. Upon plasma membrane rupture, there is a release of intracellularly segregated molecules, many of which are called *alarmins* for their ability to signal the cell damage to specific receptors on adjacent cells [31]. Obviously, n*ecrosis* is more evident in the regions more distant from tissue vessels, where pO_2_ reaches the lowest levels.

In *chronic* mild hypoxia, most cells (especially less differentiated cells and stem cells) are able to survive adapting their phenotype to the low pO_2_ [3]. This *adaptation* to hypoxia occurs through the activation of hypoxic inducible factors (HIFs) [32] and the expression of a number of HIF-1*α*-dependent genes, involved in vital pathways, such as angiogenesis (vascular endothelial growth factors or VEGFs), metabolism (glucose transporter 1 or Glut1, hexokinase II or HKII, and glycolysis) [33], inflammation (Toll-like receptors or TLRs and other receptors for alarmins, cytokines, and matrix metalloproteinases or MMPs), and repair (autophagocytosis for disposal of damaged cell components, telomerase reverse transcriptase (TERT), and stemness genes to increase the stem cell compartment to substitute death cells) [34], transforming growth factor *β* (TGF-*β*) and fibrosis pathways [33].

#### 3.2.2. Amplification and Maintenance of a Chronic IRR in Amyloidosic/Hypoxic Microenvironment

The importance of the unconventional expression of receptors for alarmins must be underlined, directly in parenchymal cells (probably *less differentiated and resident stem cells*) [35] which allows the acquisition of the proinflammatory phenotype in nonleukocytic (CD45-) cells, such as neurons, astrocytes, neuroglia, epithelial cells, and muscle cells [7]. Unconventionally, these cells express receptor for advanced glycation end products (RAGE), purinergic type 2 X7 (P2X7), Toll-like receptors, nucleotide oligomerization domain-like (NOD-like) receptors, inflammasomes, etc. [5], normally abundantly observed in activated leukocytes and endothelial cells.

As a consequence, alarmins released by necrotic cells (*β*-fibrils, high-mobility group box 1 or HMGB1, ATP/ADP, membrane debris, nucleic acids, etc.) bind to these newly expressed receptors producing an additional activation of transcription factors (NF*κ*B, STAT3, AP1, etc.) driving IRR gene transcription not only in resident inflammatory cells but also in remodeled parenchymal cells [26, 27]. The latter further activate oxidative metabolism, produce mediators and other proinflammatory cytokines, and maintain a chronic inflammatory status in the affected tissue. Unfortunately, clinical consequences of this prolonged IRR activation include cell/tissue damage, continuous repair and fibrosis with progressive deterioration of the function leading to organ insufficiency, and neuronal damages, the typical final outcome of amyloidoses [5, 7, 36].

## 4. Mechanism of Tissue Damage

A number of different mechanisms contribute to the damage of affected tissue. However, they can all be included in three categories: (a) direct toxicity of *β*-fibrils, (b) structural damage and phenotype remodeling produced by the progressive growth of *β*-aggregates, and (c) activation of natural immunity or inflammatory response, ROS production, cell damage, chronic repair, fibrosis, and functional insufficiency.

Although a general consensus on these categories is present, a number of questions are still unresolved.

*(a) Direct Toxicity of β-Fibrils*. The chemical patterns of *β*-fibrils share many characteristics with exogenous pathogen-associated molecular patterns (PAMPs) or endogenous damage-associated molecular patterns (DAMPs). Therefore, extracellular *β*-fibrils may be recognized by alarmin receptors (Toll-like receptors, RAGE, and P2X7) [37, 38] and pentraxin family members such as SAP [39]. Extracellular *β*-fibril-alarmin receptor binding can then activate NF*κ*B pathways for oxidative metabolism and apoptosis [40, 41] as well as phagocytic activity of macrophages (Figure 1(c)). On the other hand, intracellular *β*-fibrils are able to activate inflammasomes [42, 43]. Through these mechanisms, inflammation and ROS contribute substantially to the cell/tissue damage and severity of the disease.

*(b) Structural Damage and Phenotype Remodeling Associated by the Progressive Growth of β-Aggregates*. The progressive accumulation of *β*-fibrils is mainly due to their insensitivity to proteases that usually dispose of misfolded/aged proteins and their polymers [44, 45]. Although intracellular *β*-fibrils may undergo autophagocytosis, they still display a clear insensitivity to the intralysosomal acidic proteases, leading to lipofuscin accumulation that can be observed in aged and amyloidosic tissues [46]. An obvious consequence of *β*-fibril accumulation is the progressive architectural alteration of both cells and tissues with functional loss.

*(c) Activation of Inflammatory-Reparative Response*. In conclusion, as it has been underlined previously, progressive accumulation of amyloid fibrils is responsible for a local hypoxia and of a chronic long lasting inflammation, which in turn produces tissue damage leading to continuous repair, fibrosis, and organ insufficiency.

### 4.1. Putative Therapeutic Targets

Amyloidoses have an urgent need for new and effective therapeutic strategies. A better definition of the pathogenetic scenario here presented might suggest more precise and rational targets, changing the present disappointing and malignant outcomes of these diseases.

**Figure 4:**
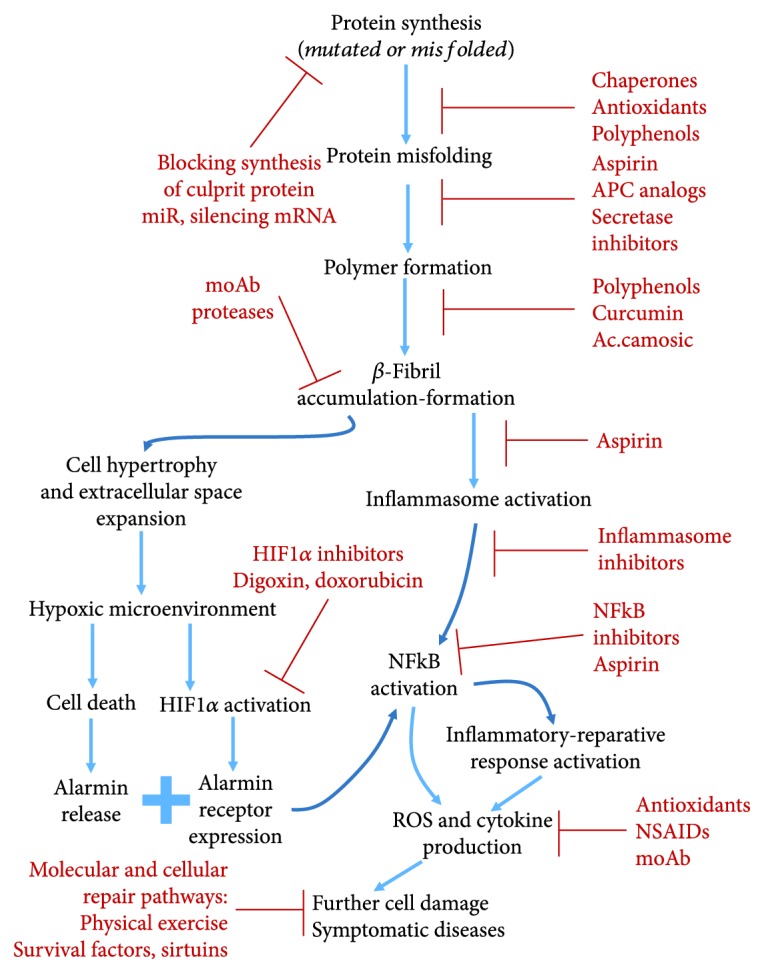
*β*-Aggregate formation and sequence of the tissue/organ damage. The possible therapeutic targets and the potential pharmacological agents are indicated in red color (see also text).

Three main approaches can be pursued: (a) control the synthesis of misfolded *β*-sheet-rich proteins and prevent/correct their misfolding; (b) inhibit polymer/fibril formation and accumulation and/or favor their clearance; and (c) inhibit and modulate HIF-1*α*/NF*κ*B axis activation, limiting or repairing the damage associated to the *β*-fibrils.  Figure 4 summarizes these approaches.

(a) The use of specific microRNA [47, 48] and RNA silencing [49] blocks/reduces the synthesis of the culprit protein and ameliorates mitochondrial respiration, synaptic function, and clinical symptoms. Recently, it has been shown [50] that recombinant APC (activated protein C, a plasma protease) or its analogs, inhibiting the neuronal *β*-secretase, may strongly slow or block the generation of *β*-amyloid protein protecting from Alzheimer's disease or slowing its progression [51, 52]

(b) Using chaperones [53, 54], small molecules [54], and aspirin [55] slows or inhibits polymer formation [50, 55, 56] and *β*-fibril aggregation and accumulation, preventing the disease and attenuating its progression and symptoms. In cellular and animal models, *aspirin* has been shown to be highly effective in inhibiting polymer formation and *β*-fibril aggregation with *in vivo* reduction of the incidence of Alzheimer's and Parkinson's diseases. Although the mechanism(s) is still unclear, it has been demonstrated that aspirin is able to donate its acetyl group to the culprit proteins, increasing their acetylation and reducing the tendency of phosphorylation. This appears to be a common mechanism for inhibiting polymer and *β*-fibril formation in all different *β*-fibrillosis [55]. Aspirin may, as well, contribute with other beneficial mechanisms, such as inhibition of NF*κ*B and cyclooxygenase, reducing the damaging impact of the inflammatory-reparative response (see below)

(c) Modulating HIF-1*α* and hypoxia adaptation [3, 4, 53] can represent a new strategy to block or limit the damage in tissues accumulating *β*-fibrils. A long list of potential HIF-1*α* inhibitors is now available, such as digoxin, acriflavine, doxorubicin, and chetomin [57–59]

(d) The utilization of NF*κ*B inhibitors, anti-inflammatory drugs, and anti-inflammasome agents is aimed at modulating and inhibiting chronic inflammation and ROS production responsible for continuous cell damage, repair, and fibrosis [31, 60]

(e) Microbiota and nutrients may be in many ways involved in the pathogenesis of *β*-fibrillosis, including neurodegenerative diseases such as Alzheimer's and Parkinson's disease. Surprisingly, an intense gut-brain cross-talk has been evidenced, suggesting an important therapeutic role of the maintenance of intestinal microbiome equilibrium in preventing neurodegenerative diseases [61–63]. As a consequence, natural nutrients [64] such as curcumin and in particular vanillin, a degradation product of curcumin, have been shown to reduce the formation of advanced glycation end products (AGEs) [65]. Therefore, it is conceivable that they may also be used to modify microbiota with the goal to prevent, slow, and ameliorate beta-fibrillosis [64]

(f) Recently, *physical exercise* has been shown to be able to prevent Alzheimer's disease and substantially slow its progression. Mechanisms are unclear and controversial, but it seems that endocrine and metabolic effects associated with physical activity may be responsible for these beneficial effects. In particular, the release of survival factors, such as BDGF (neurons), IGF-1 (systemic or organ-specific release), and testosterone, and the activation of survival/repairing pathways, such as sirtuin-dependent transcription factors, are able to reduce apoptosis and to repair abnormal cell components, rescuing sublethally damaged postmitotic cells, such as neurons [66], myocardiocytes, skeletal muscle, and hepatocytes [67]

## 5. Conclusions

Clinically, amyloidoses are chronic progressive degenerative diseases leading to severe and irreversible insufficiency/failure of the involved organ/tissue. Even if genetic or acquired protein misfolding represents the primary trigger of the disease, during the pathogenetic sequence and progression, other pathophysiological responses are activated that strongly contribute to the damage production [68]. In particular, activation of the HIF-1*α*/NF*κ*B axis by local hypoxia produces a proinflammatory remodeling of the affected tissue, explaining the final fibrosis and organ/tissue failure. Therefore, hypoxia and inflammation must be taken into consideration as potential targets for more rational and effective therapies aimed not only at preventing the formation and accumulation of *β*-fibrils and/or at increasing their clearance from deposits but more importantly at blocking/reducing the damage associated with the chronic inflammatory-reparative response.

## Figures and Tables

**Table 1:** Common molecular features of amyloidosis.

Molecular features	Description and mechanisms	References
Misfolded proteins	Misfolding of *β*-sheet-rich proteins, such as amyloid *β*-protein tau, tau, *α*-synuclein, and prion protein (PrP^sc^)	[1, 24]
Polymers/protofibrils	Formation of intra/extracellular polymers with antiparallel *β*-sheet-rich proteins or protofibrils	[69]
*β*-Fibrils at optical microscopy (OM)	*β*-Fibrils show green birefringence at polarized OM after red Congo staining	[2]
*β*-Fibrils at TEM	*β*-Fibrils are linear, 8-12 nm in diameter, and interact with EM extracellular matrix molecules	[1, 2, 12]
*β*-Fibril physicochemistry	Linear, rigid, nonbranching, and protease-resistant polymers, probably interacting with extracellular matrix proteins	[12, 70]
*β*-Fibril protein composition	*β*-Fibril proteins are heterogeneous in their origin and composition, depending on the cell type involved	[12]
*β*-Fibril passing in the blood	*β*-Fibrils associate with SAP (serum amyloid protein)	[1, 2]
Large aggregates and deposits of *β*-fibrils	Formations of aggregates and deposits of rigid, stable, and protease-resistant *β*-fibrils, containing 15% of SAP, localized in the extracellular space, mostly around the vessel	[71]

**Table 2:** Common clinical features of amyloidosic diseases.

Clinical features	Description and mechanisms	References
Familial	Mutations of the involved protein, strictly influencing misfolding and conformational instability, such as transthyretin in familial amyloidosic polyneuropathy	[72]
Acquired	Conditions presenting abnormal/toxic production and posttransductional misfolding of culprit proteins, such as plasmocytoma (light chains) or chronic inflammatory diseases (SAA) or haemodialysis-related amyloidosis (*β*_2_-microglobulin)	[1, 73]
Localized	Organ-limited amyloidosis in which *β*-fibrils and polymers may became resident, almost stably, in the extracellular space around the cells producing the misfolded protein (see text)	[1]
Systemic	Bulk production and secretion of culprit protein in the extracellular space; protein entering the vessel lumen may be evidenced in blood plasma by a typical electrophoretic peak; accumulation in the extracellular space of distant tissues around the organism (liver, kidney, myocardium, lungs, brain, etc.), such as primary systemic amyloidosis (Ig light chains) and senile systemic amyloidosis (nonmutated transthyretin)	[1]
Progressive	The rate of amyloid accumulation depends not only on the rate of synthesis and on insensitivity to the extracellular proteases but also on the early start and duration length of disease.	[13, 23–25]
False hypertrophy	Both producing cells and accumulating tissues/organs increase their volume at various sizes, depending on the degree of progression.	[23, 74]
Systemic inflammation	A low-degree inflammation is constantly present in a patient bearing amyloidosis. Its intensity level is determined by the strength of the activation mechanisms (see text) and by the nature of the involved protein.	[5, 23, 75]
Hypoxia	There are a few specific studies demonstrating that the space accumulating the amyloid substance is actually hypoxic. However, an accurate evaluation of the distance between the vessels and the peripheral parenchymal cells shows that frequently this is larger than 200 *μ*m, which is the diffusion limit of gas such as oxygen and carbon dioxide.	[3, 76]
